# The genome sequence of a hoverfly, *Pocota personata *(Harris, 1780)

**DOI:** 10.12688/wellcomeopenres.20501.1

**Published:** 2023-11-23

**Authors:** Steven Falk, Katie J. Woodcock, University of Oxford and Wytham Woods Genome Acquisition Lab, Darwin Tree of Life Barcoding collective, Wellcome Sanger Institute Tree of Life Management, Samples and Laboratory team, Wellcome Sanger Institute Scientific Operations: Sequencing Operations, Wellcome Sanger Institute Tree of Life Core Informatics team, Tree of Life Core Informatics collective, Darwin Tree of Life Consortium

**Affiliations:** 1 Independent Researcher, Kenilworth, England, UK; 2 Tree of Life, Wellcome Sanger Institute, Hinxton, England, UK

**Keywords:** Pocota personata, hoverfly, genome sequence, chromosomal, Diptera

## Abstract

We present a genome assembly from an individual male *Pocota personata* (a hoverfly; Arthropoda; Insecta; Diptera; Syrphidae). The genome sequence is 845.2 megabases in span. Most of the assembly is scaffolded into 5 chromosomal pseudomolecules, including the X sex chromosome. The mitochondrial genome has also been assembled and is 16.04 kilobases in length.

## Species taxonomy

Eukaryota; Metazoa; Eumetazoa; Bilateria; Protostomia; Ecdysozoa; Panarthropoda; Arthropoda; Mandibulata; Pancrustacea; Hexapoda; Insecta; Dicondylia; Pterygota; Neoptera; Endopterygota; Diptera; Brachycera; Muscomorpha; Eremoneura; Cyclorrhapha; Aschiza; Syrphoidea; Syrphidae; Eristalinae; Milesiini; *Pocota*; *Pocota personata* (Harris, 1780) (NCBI:txid2867115).

## Background

*Pocota personata* (Harris, 1780) is a European woodland hoverfly species, in the UK records are predominantly associated with wooded areas rich in dead and decaying trees in southern England, though it has been found as far north as North Yorkshire and County Durham ( Ball & Morris, 2000; Ball & Morris, 2015; Stubbs & Falk, 2002; van Veen, 2014). It is described as a rare hoverfly throughout Europe with the majority of UK records existing between the months of April to August and a peak in numbers occurring during mid to late May ( Ball & Morris, 2000; Ball & Morris, 2015; Stubbs & Falk, 2002). As larvae, *P. personata* are dark in colour and preferentially occupy elevated beech and poplar tree rot holes, activities relating to ‘forest hygiene’ including clearing of rotting logs and dead trees and filling of rot holes have been common practice in the past and may have impacted this species ( Gandy, 2019; Rotheray, 1993; Stubbs & Falk, 2002). As adults *P. personata* are stocky bumblebee mimics, known for acoustic and behavioural mimicry through production of a distinctively loud buzz as well as repeated lifting of a hind leg if disturbed ( Ball & Morris, 2015; Gandy, 2019; Stubbs & Falk, 2002). Adults possess two unique recognisable features setting them aside from other Syrphid bumblebee mimics, firstly a disproportionately small head, measuring approximately half the thorax width and additionally the presence of prominent bright yellow hairs on the thorax and abdomen ( Ball & Morris, 2015). Flower visitation appears to be an occasional behaviour in *P. personata*, though it has been spotted visiting umbellifers, hawthorn and apple trees during the late afternoon and early evening ( Ball & Morris, 2015; Stubbs & Falk, 2002). The completed genome sequence for *Pocota personata* is an important resource in aiding the future study of this scarce and poorly understood hoverfly species.

## Genome sequence report

**Figure 1.**
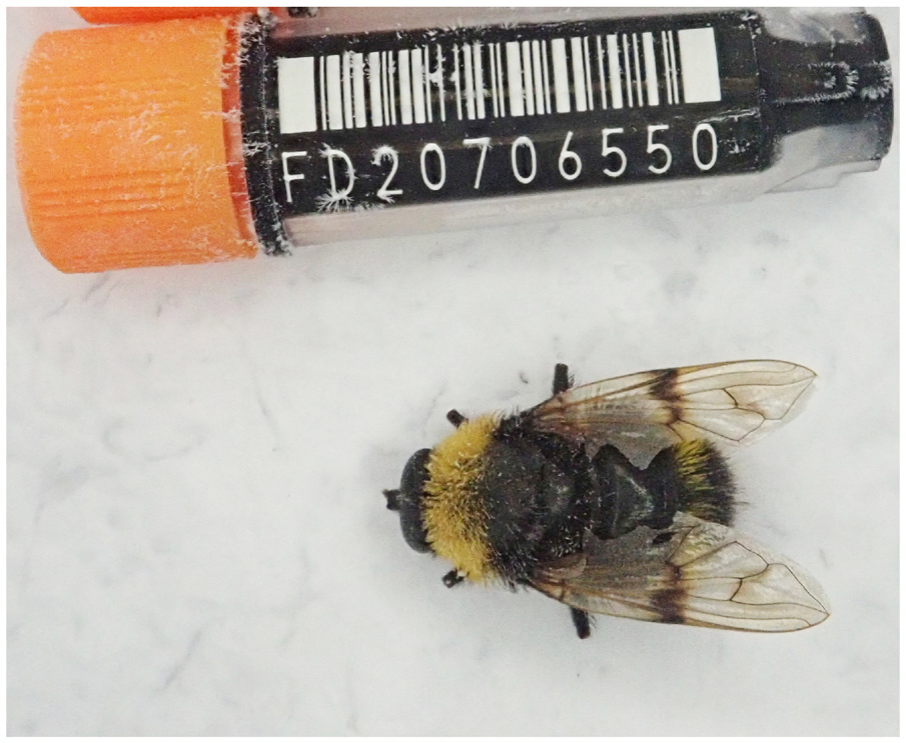
Photograph of the *Pocota personata* (idPocPers1) specimen used for genome sequencing.

The genome was sequenced from one male *Pocota personata* ( Figure 1) collected from Wytham Woods, Oxfordshire, UK (51.76, –1.33). A total of 30-fold coverage in Pacific Biosciences single-molecule HiFi long reads was generated. Primary assembly contigs were scaffolded with chromosome conformation Hi-C data. Manual assembly curation corrected 108 missing joins or mis-joins and removed one haplotypic duplication, reducing the scaffold number by 22.22%, and increasing the scaffold N50 by 158.88%.

**Figure 2.**
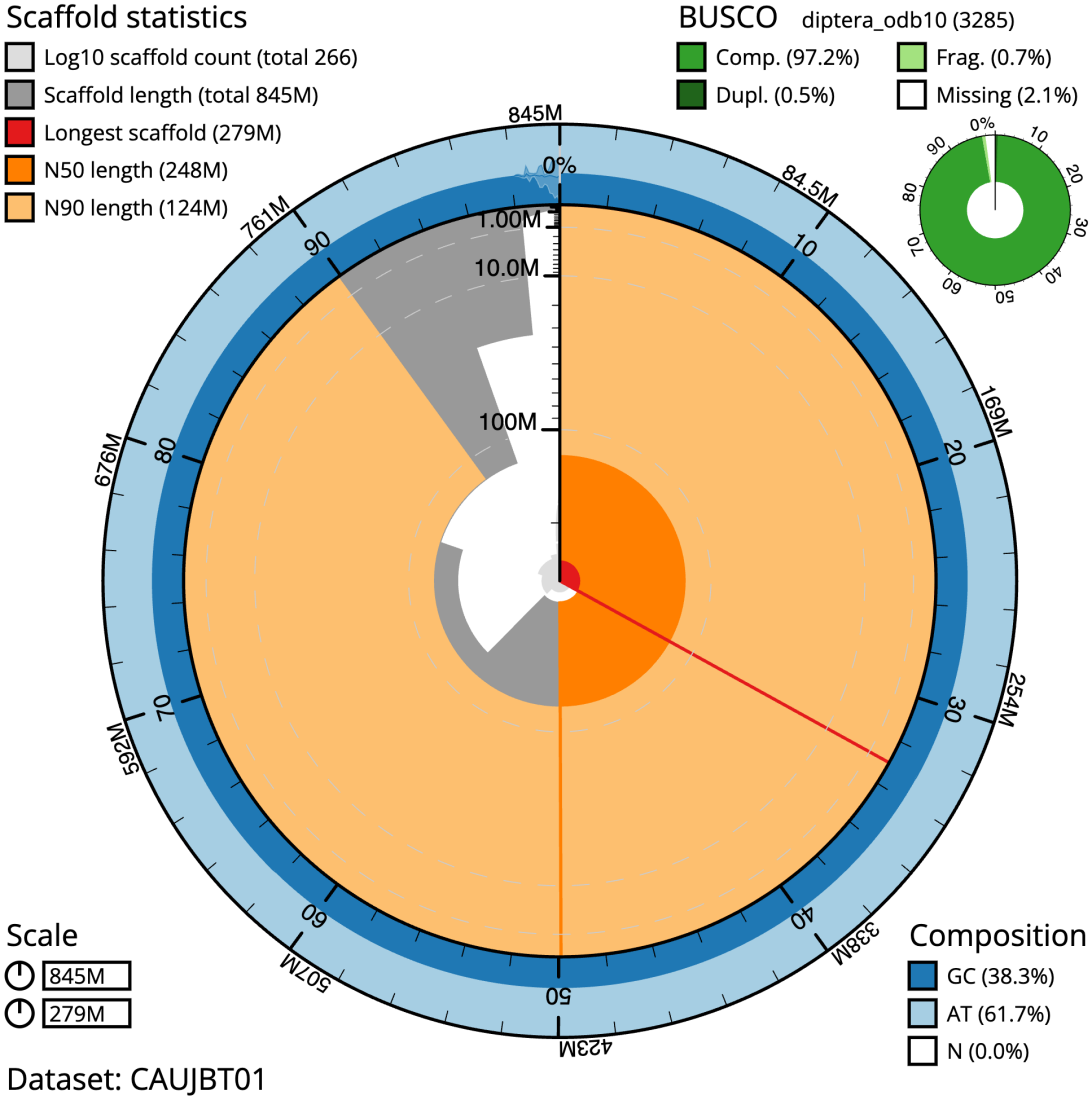
Genome assembly of *Pocota personata*, idPocPers1.1: metrics. The BlobToolKit Snailplot shows N50 metrics and BUSCO gene completeness. The main plot is divided into 1,000 size-ordered bins around the circumference with each bin representing 0.1% of the 845,253,571 bp assembly. The distribution of scaffold lengths is shown in dark grey with the plot radius scaled to the longest scaffold present in the assembly (279,389,706 bp, shown in red). Orange and pale-orange arcs show the N50 and N90 scaffold lengths (247,560,496 and 123,742,089 bp), respectively. The pale grey spiral shows the cumulative scaffold count on a log scale with white scale lines showing successive orders of magnitude. The blue and pale-blue area around the outside of the plot shows the distribution of GC, AT and N percentages in the same bins as the inner plot. A summary of complete, fragmented, duplicated and missing BUSCO genes in the diptera_odb10 set is shown in the top right. An interactive version of this figure is available at https://blobtoolkit.genomehubs.org/view/Pocota%20personata/dataset/CAUJBT01/snail.

**Figure 3.**
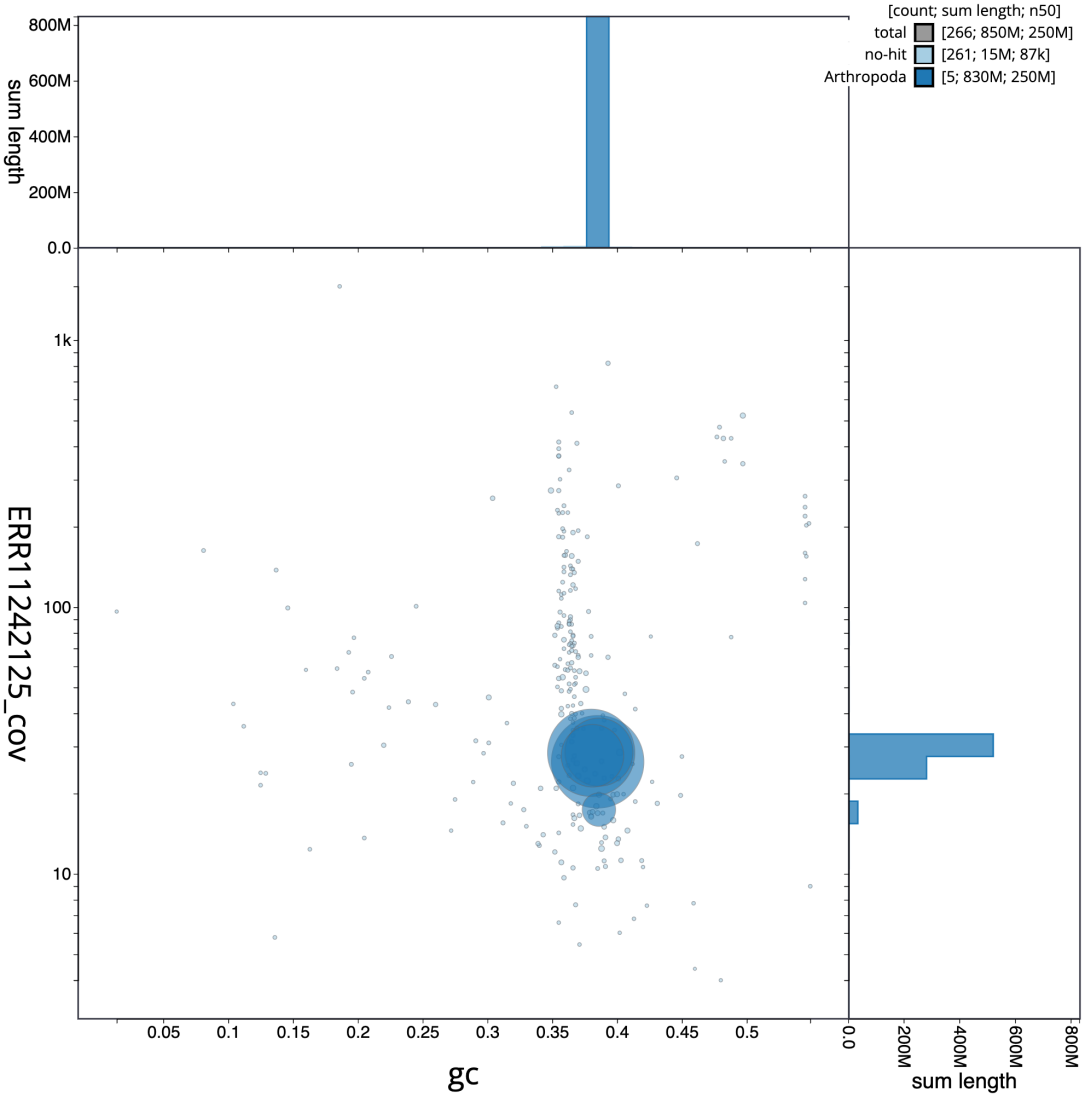
Genome assembly of *Pocota personata*, idPocPers1.1: BlobToolKit GC-coverage plot. Scaffolds are coloured by phylum. Circles are sized in proportion to scaffold length. Histograms show the distribution of scaffold length sum along each axis. An interactive version of this figure is available at https://blobtoolkit.genomehubs.org/view/Pocota%20personata/dataset/CAUJBT01/blob.

**Figure 4.**
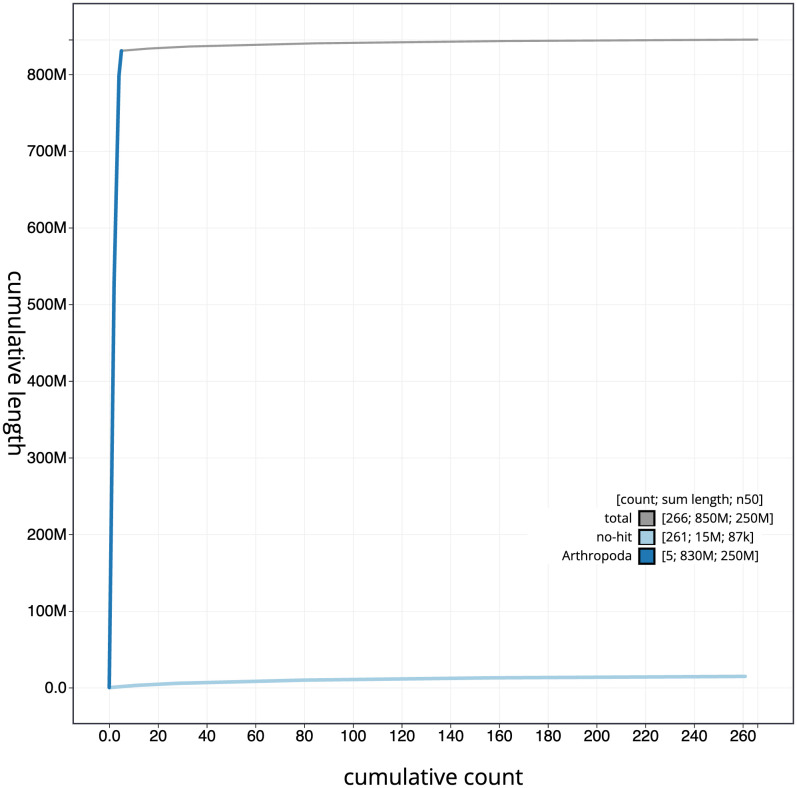
Genome assembly of *Pocota personata*, idPocPers1.1: BlobToolKit cumulative sequence plot. The grey line shows cumulative length for all scaffolds. Coloured lines show cumulative lengths of scaffolds assigned to each phylum using the buscogenes taxrule. An interactive version of this figure is available at https://blobtoolkit.genomehubs.org/view/Pocota%20personata/dataset/CAUJBT01/cumulative.

**Figure 5.**
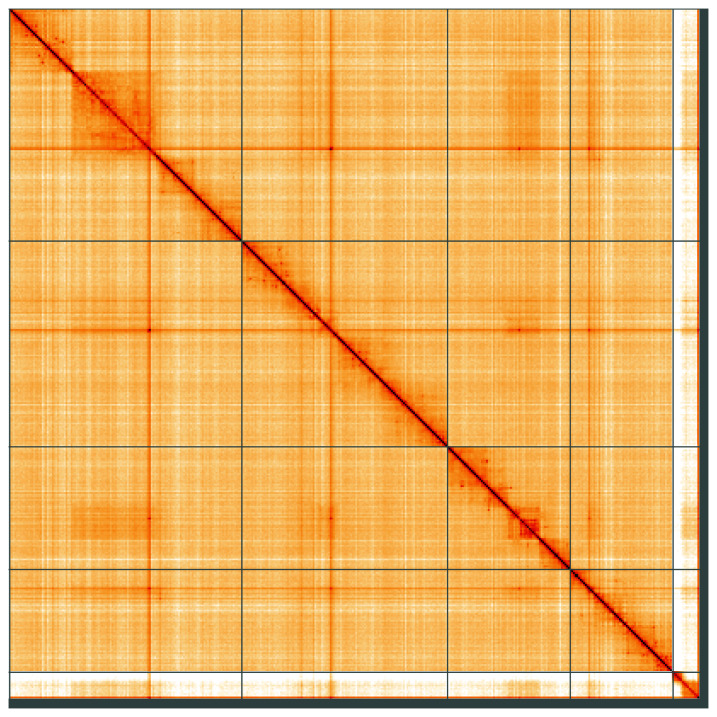
Genome assembly of *Pocota personata*, idPocPers1.1: Hi-C contact map of the idPocPers1.1 assembly, visualised using HiGlass. Chromosomes are shown in order of size from left to right and top to bottom. An interactive version of this figure may be viewed at https://genome-note-higlass.tol.sanger.ac.uk/l/?d=Ikse1PuTTceSUbllW6lsUQ.

The final assembly has a total length of 845.2 Mb in 265 sequence scaffolds with a scaffold N50 of 247.6 Mb ( Table 1). The snailplot in Figure 2 provides a summary of the assembly statistics, while the distribution of assembly scaffolds on GC proportion and coverage is shown in Figure 3. The cumulative assembly plot in Figure 4 shows curves for subsets of scaffolds assigned to different phyla. Most (98.27%) of the assembly sequence was assigned to 5 chromosomal-level scaffolds, 4 autosomes and the X sex chromosome. Chromosome X was identified by read coverage statistics. No Y was identified. The species is thought to be XO. Chromosome-scale scaffolds confirmed by the Hi-C data are named in order of size ( Figure 5; Table 2). While not fully phased, the assembly deposited is of one haplotype. Contigs corresponding to the second haplotype have also been deposited. The mitochondrial genome was also assembled and can be found as a contig within the multifasta file of the genome submission.

**Table 1.** Genome data for *Pocota personata*, idPocPers1.1.

Project accession data		
Assembly identifier	idPocPers1.1	
Species	*Pocota personata*	
Specimen	idPocPers1	
NCBI taxonomy ID	2867115	
BioProject	PRJEB61333	
BioSample ID	SAMEA10167087	
Isolate information	idPocPers1, male: abdomen (DNA), head (Hi-C data)	
Assembly metrics *		*Benchmark*
Consensus quality (QV)	61.0	*≥ 50*
*k*-mer completeness	100%	*≥ 95%*
BUSCO **	C:97.2%[S:96.7%,D:0.5%],F:0.7%,M:2.1%,n:3,285	*C ≥ 95%*
Percentage of assembly mapped to chromosomes	98.27%	*≥ 95%*
Sex chromosomes	X chromosome	*localised homologous pairs*
Organelles	Mitochondrial genome assembled	*complete single alleles*
Raw data accessions		
PacificBiosciences SEQUEL II	ERR11242125	
Hi-C Illumina	ERR11242538	
Genome assembly		
Assembly accession	GCA_963082735.1	
*Accession of alternate haplotype*	GCA_963083005.1	
Span (Mb)	845.2	
Number of contigs	907	
Contig N50 length (Mb)	2.9	
Number of scaffolds	265	
Scaffold N50 length (Mb)	247.6	
Longest scaffold (Mb)	279.4	

* Assembly metric benchmarks are adapted from column VGP-2020 of “Table 1: Proposed standards and metrics for defining genome assembly quality” from ( Rhie *et al.*, 2021). ** BUSCO scores based on the diptera_odb10 BUSCO set using v5.3.2. C = complete [S = single copy, D = duplicated], F = fragmented, M = missing, n = number of orthologues in comparison. A full set of BUSCO scores is available at https://blobtoolkit.genomehubs.org/view/Pocota%20personata/dataset/CAUJBT01/busco

**Table 2.** Chromosomal pseudomolecules in the genome assembly of *Pocota personata*, idPocPers1.

INSDC accession	Chromosome	Length (Mb)	GC%
OY720429.1	1	279.39	38.5
OY720430.1	2	247.56	38.0
OY720431.1	3	147.65	38.5
OY720432.1	4	123.74	38.0
OY720433.1	X	32.32	38.5
OY720434.1	MT	0.02	19.5

The estimated Quality Value (QV) of the final assembly is 61.0 with *k*-mer completeness of 100%, and the assembly has a BUSCO v5.3.2 completeness of 97.2% (single = 96.7%, duplicated = 0.5%), using the diptera_odb10 reference set ( *n* = 3,285).

Metadata for specimens, barcode results, spectra estimates, sequencing runs, contaminants and pre-curation assembly statistics are given at https://links.tol.sanger.ac.uk/species/2867115.

## Methods

### Sample acquisition and nucleic acid extraction

A male *Pocota personata* (specimen ID Ox001535, ToLID idPocPers1) was netted in Wytham Woods, Oxfordshire (biological vice-country Berkshire), UK (latitude 51.76, longitude –1.33) on 2021-05-31. The specimen was collected and identified by Steven Falk (University of Oxford) and preserved on dry ice.

High molecular weight (HMW) DNA was extracted at the Tree of Life laboratory, Wellcome Sanger Institute (WSI), following a sequence of core procedures: sample preparation; sample homogenisation; HMW DNA extraction; DNA fragmentation; and DNA clean-up. The idPocPers1sample was weighed and dissected on dry ice (as per the protocol https://dx.doi.org/10.17504/protocols.io.x54v9prmqg3e/v1). The abdomen of the idPocPers1 sample was homogenised using a Nippi Powermasher fitted with a BioMasher pestle, following the protocol at https://dx.doi.org/10.17504/protocols.io.5qpvo3r19v4o/v1. DNA was extracted by means of the HMW DNA Extraction: Automated MagAttract protocol ( https://dx.doi.org/10.17504/protocols.io.kxygx3y4dg8j/v1). HMW DNA was sheared into an average fragment size of 12–20 kb in a Megaruptor 3 system with speed setting 30, following the HMW DNA Fragmentation: Diagenode Megaruptor ^®^3 for PacBio HiFi protocol ( https://dx.doi.org/10.17504/protocols.io.8epv5x2zjg1b/v1). Sheared DNA was purified using Manual solid-phase reversible immobilisation (SPRI) (protocol at https://dx.doi.org/10.17504/protocols.io.kxygx3y1dg8j/v1). In brief, the method employs a 1.8X ratio of AMPure PB beads to sample to eliminate shorter fragments and concentrate the DNA. The concentration of the sheared and purified DNA was assessed using a Nanodrop spectrophotometer and Qubit Fluorometer and Qubit dsDNA High Sensitivity Assay kit. Fragment size distribution was evaluated by running the sample on the FemtoPulse system.

Protocols developed by the Tree of Life laboratory are publicly available on protocols.io: https://dx.doi.org/10.17504/protocols.io.8epv5xxy6g1b/v1.

### Sequencing

Pacific Biosciences HiFi circular consensus DNA sequencing libraries were constructed according to the manufacturers’ instructions. DNA sequencing was performed by the Scientific Operations core at the WSI on a Pacific Biosciences SEQUEL II instrument. Hi-C data were also generated from head tissue of idPocPers1 using the Arima2 kit and sequenced on the Illumina NovaSeq 6000 instrument.

### Genome assembly, curation and evaluation

Assembly was carried out with Hifiasm ( Cheng *et al.*, 2021) and haplotypic duplication was identified and removed with purge_dups ( Guan *et al.*, 2020). The assembly was then scaffolded with Hi-C data ( Rao *et al.*, 2014) using YaHS ( Zhou *et al.*, 2023). The assembly was checked for contamination and corrected as described previously ( Howe *et al.*, 2021). Manual curation was performed using HiGlass ( Kerpedjiev *et al.*, 2018) and Pretext ( Harry, 2022). The mitochondrial genome was assembled using MitoHiFi ( Uliano-Silva *et al.*, 2023), which runs MitoFinder ( Allio *et al.*, 2020) or MITOS ( Bernt *et al.*, 2013) and uses these annotations to select the final mitochondrial contig and to ensure the general quality of the sequence.

A Hi-C map for the final assembly was produced using bwa-mem2 ( Vasimuddin *et al.*, 2019) in the Cooler file format ( Abdennur & Mirny, 2020). To assess the assembly metrics, the *k*-mer completeness and QV consensus quality values were calculated in Merqury ( Rhie *et al.*, 2020). This work was done using Nextflow ( Di Tommaso *et al.*, 2017) DSL2 pipelines “sanger-tol/readmapping” ( Surana *et al.*, 2023a) and “sanger-tol/genomenote” ( Surana *et al.*, 2023b). The genome was analysed within the BlobToolKit environment ( Challis *et al.*, 2020) and BUSCO scores ( Manni *et al.*, 2021; Simão *et al.*, 2015) were calculated.

Table 3 contains a list of relevant software tool versions and sources.

**Table 3.** Software tools: versions and sources.

Software tool	Version	Source
BlobToolKit	4.2.1	https://github.com/blobtoolkit/blobtoolkit
BUSCO	5.3.2	https://gitlab.com/ezlab/busco
Hifiasm	0.16.1-r375	https://github.com/chhylp123/hifiasm
HiGlass	1.11.6	https://github.com/higlass/higlass
Merqury	MerquryFK	https://github.com/thegenemyers/MERQURY.FK
MitoHiFi	3	https://github.com/marcelauliano/MitoHiFi
PretextView	0.2	https://github.com/wtsi-hpag/PretextView
purge_dups	1.2.5	https://github.com/dfguan/purge_dups
sanger-tol/ genomenote	v1.0	https://github.com/sanger-tol/genomenote
sanger-tol/ readmapping	1.1.0	https://github.com/sanger-tol/readmapping/tree/1.1.0
YaHS	1.2a.2	https://github.com/c-zhou/yahs

### Wellcome Sanger Institute – Legal and Governance

The materials that have contributed to this genome note have been supplied by a Darwin Tree of Life Partner. The submission of materials by a Darwin Tree of Life Partner is subject to the **‘Darwin Tree of Life Project Sampling Code of Practice’**, which can be found in full on the Darwin Tree of Life website here. By agreeing with and signing up to the Sampling Code of Practice, the Darwin Tree of Life Partner agrees they will meet the legal and ethical requirements and standards set out within this document in respect of all samples acquired for, and supplied to, the Darwin Tree of Life Project. 

Further, the Wellcome Sanger Institute employs a process whereby due diligence is carried out proportionate to the nature of the materials themselves, and the circumstances under which they have been/are to be collected and provided for use. The purpose of this is to address and mitigate any potential legal and/or ethical implications of receipt and use of the materials as part of the research project, and to ensure that in doing so we align with best practice wherever possible. The overarching areas of consideration are:

•   Ethical review of provenance and sourcing of the material

•   Legality of collection, transfer and use (national and international)

Each transfer of samples is further undertaken according to a Research Collaboration Agreement or Material Transfer Agreement entered into by the Darwin Tree of Life Partner, Genome Research Limited (operating as the Wellcome Sanger Institute), and in some circumstances other Darwin Tree of Life collaborators.

## Data Availability

European Nucleotide Archive: *Pocota personata*. Accession number PRJEB61333; https://identifiers.org/ena.embl/PRJEB61333 ( Wellcome Sanger Institute, 2023). The genome sequence is released openly for reuse. The *Pocota personata* genome sequencing initiative is part of the Darwin Tree of Life (DToL) project. All raw sequence data and the assembly have been deposited in INSDC databases. Raw data and assembly accession identifiers are reported in Table 1.

## Author information

Members of the University of Oxford and Wytham Woods Genome Acquisition Lab are listed here: https://doi.org/10.5281/zenodo.7125292.

Members of the Darwin Tree of Life Barcoding collective are listed here: https://doi.org/10.5281/zenodo.4893703.

Members of the Wellcome Sanger Institute Tree of Life Management, Samples and Laboratory team are listed here: https://doi.org/10.5281/zenodo.10066175.

Members of Wellcome Sanger Institute Scientific Operations: Sequencing Operations are listed here: https://doi.org/10.5281/zenodo.10043364.

Members of the Wellcome Sanger Institute Tree of Life Core Informatics team are listed here: https://doi.org/10.5281/zenodo.10066637.

Members of the Tree of Life Core Informatics collective are listed here: https://doi.org/10.5281/zenodo.5013541.

Members of the Darwin Tree of Life Consortium are listed here: https://doi.org/10.5281/zenodo.4783558.
