# Supplementary material for: The genome sequence of a hoverfly, Pocota personata (Harris, 1780)
Source: Wellcome Open Res. 2023 Nov 23;8:546. [Version 1] doi: 10.12688/wellcomeopenres.20501.1 (PMC11259775; doi:10.12688/wellcomeopenres.20501.1)
Supplement: Peer review history

# Reviewer report: Reviewer response for version 1

**DOI:** 10.21956/wellcomeopenres.22694.r86846

**Published:** 2024-07-19

**Authors:** Pablo Aguado-Aranda

**Affiliations:** 1 University of Alicante, Alicante, Spain

First of all, I would like to congratulate the authors of this publication for their contribution to this topic. Special mention should be made of the background section which summarises the most relevant data on the species under study, my compliments to the authors.

 My comments on this manuscript are simply suggestions and small changes. For example, I would recommend adding the authorship of the species also in the abstract. Regarding the methodology, I should avoid repeating information on data collection (locality, coordinates and date) of the specimen, as it is mentioned twice in the text: (1) at the beginning of the section "Genome sequence report" and (2) in the subsection "Sample acquisition and nucleic acid extraction". Regarding this subsection, I would recommend replacing the links to the reference of each of the protocols and referencing them in the text as the rest of the references.

 Finally, as in previous reviews, as a reviewer I would like to point out that my knowledge and experience in whole genome sequencing procedures is very limited. Therefore, I suggest the editorial team to take this into account and consider other researchers with more experience in these topics for the review of this part.

Are sufficient details of methods and materials provided to allow replication by others?

Yes

Is the rationale for creating the dataset(s) clearly described?

Yes

Are the datasets clearly presented in a useable and accessible format?

Yes

Are the protocols appropriate and is the work technically sound?

Yes

Reviewer Expertise:

Taxonomy and systematics of Syrphidae

I confirm that I have read this submission and believe that I have an appropriate level of expertise to confirm that it is of an acceptable scientific standard.

# Reviewer report: Reviewer response for version 1

**DOI:** 10.21956/wellcomeopenres.22694.r77296

**Published:** 2024-04-16

**Authors:** Annabel Whibley

**Affiliations:** 1 The University of Auckland, Auckland, Auckland, New Zealand; 2 Bragato Research Institute, Lincoln, New Zealand

Falk, Woodcock and colleagues report the sequencing and assembly of the genome of the hoverfly, *Pocota personata. *The genomic resources produced are high quality and will be of value to the scientific community.  The assembly and report follow carefully developed DToL workflows and templates and these comprehensively capture metadata for software versions and assembly properties.

 I have just minor questions/comments (the second of which I have also noted in a another report I have very recently reviewed).

 (1) The intra-chromosome signals on contact map are relatively weak (compared to “typical” plots). Are there biological or technical reasons why this might be the case?

 (2) Can I query the bead ratio stated in the methods *“In brief, the method uses a 1.8X ratio of AMPure PB beads to sample to eliminate shorter fragments and concentrate the DNA.”*  In my understanding, and from the guidelines on protocol.io, to eliminate smaller fragments, a lower ratio of beads to sample would be used. Should this be 0.6x?

Are sufficient details of methods and materials provided to allow replication by others?

Yes

Is the rationale for creating the dataset(s) clearly described?

Yes

Are the datasets clearly presented in a useable and accessible format?

Yes

Are the protocols appropriate and is the work technically sound?

Yes

Reviewer Expertise:

Genomics, Evolution, Bioinformatics

I confirm that I have read this submission and believe that I have an appropriate level of expertise to confirm that it is of an acceptable scientific standard.
